# Medicinal plant genomics

**DOI:** 10.1186/s12864-023-09542-8

**Published:** 2023-08-01

**Authors:** Christian Siadjeu, Boas Pucker

**Affiliations:** 1 Prinzessin Therese Von Bayern Chair of Systematics, Biodiversity and Evolution of Plants, Ludwig Maximilian University Munich, 80638 Munich, Germany; 2 Plant Biotechnology and Bioinformatics, Institute of Plant Biology & BRICS, TU Braunschweig, 38106 Braunschweig, Germany

## Abstract

Recent developments in plant genomics have enabled a comprehensive analysis of the medicinal potential of plants based on their gene repertoire. Genes of biosynthesis pathways can be discovered through comparative genomics and through integration of transcriptomic data. Data-driven discovery of specialized metabolites could accelerate research.

The emergence and rapid development of long read sequencing technologies has been revolutionizing the field of genomics by substantially increasing assembly continuity [1]. An ongoing competition between Oxford Nanopore Technologies and Pacific Biosciences has led to rapid and continuous releases of innovations. Furthermore, the availability of portable and affordable sequencing instruments, as well as reduced sequencing costs, has democratized genomics and substantially increased the number of scientists who are applying genomic approaches and thus led to an increase in genome sequencing projects [2]. Now, studies are not only limited to the genomes of economically important crops, but also include the genomes of medicinal plants. Additional large omics data sets are often analyzed in an integrated way based on sophisticated bioinformatics tools. Indeed, a genomic sequence can advance projects that are aiming to unravel a certain biosynthesis pathway. While the generation of an almost complete genome sequence based on highly accurate long reads is becoming a routine task, the generation of the corresponding high-quality structural annotation is a growing challenge. RNA-seq and direct RNA sequencing can provide external hints to support this structural annotation process but revealing the functions of genes has become a bottleneck on the route to fully exploiting the potential of plants.

Besides food production and ecological roles, plants are also amazing chemists that synthesize a plethora of specialized metabolites. As recently reviewed, the precise number of different metabolites produced by plants is unknown, but estimates go up to about one million [3]. Many of these metabolites are of high value for humans. For example, plant pigments like anthocyanins, betalains, and carotenoids can be used as food colorants [4]. In addition to a nice visual appearance, important nutritional roles or even medicinal properties have been reported for these compounds. Flavonoids, including anthocyanins, are well known for their antioxidant properties which can protect various cell components against reactive oxygen species under stress conditions. Consequently, an anthocyanin-rich diet might help to prevent the onset or development of certain diseases. As flavonoids are present across land plants, there is a huge diversity of different derivatives that occur in specific evolutionary lineages. Specific types of flavonoids have already been considered as drug candidates. For example, the biosynthesis of the α-amylase inhibitor montbretin A in *Crocosmia x crocosmiiflora* was elucidated and has thus enabled its heterologous production [5].

Alkaloids, such as colchicine, have long been used to both prevent and treat gout attacks, a severe inflammation linked to arthritis. Despite having been utilized for more than a thousand years, the specific mechanism of colchicine production is still not fully understood. Recently, a draft genome sequence of *Cremastra appendiculata* (D. Don) has facilitated the identification of candidate genes associated with colchicine biosynthesis, highlighting the predominant role of an O-methyltransferase [6].

Similarly, terpenes are bioactive compounds with significant medicinal properties. Artemisinin is an endoperoxide sesquiterpene lactone produced in the glandular trichomes of *Artemisia annua*. Genomic sequencing, combined with transcriptome analysis, has revealed novel genes involved in artemisinin biosynthesis, and demonstrated that overexpressing the transcription factor *AaMYB2* can significantly enhance the content of artemisinin and dihydroartemisinic acid in transgenic *A. annua* lines [7]. Particularly relevant in the context of specialized metabolites in plants are biosynthetic gene clusters that gained attention in recent years [8]. The products synthesized by the enzymes encoded by these biosynthetic gene clusters are often associated with responses against pathogens.

A high-quality genome sequence with an accurate structural annotation can serve as the basis for the identification of biosynthesis genes and information about the individual functions of genes can be transferred from other plant species based on the assumption that orthologs have the same functions. This enables a high-throughput annotation workflow. Researchers can thus access a wealth of information about individual genes through genome browsers which facilitates the search for promising plant enzymes. Additional hints for the identification of biosynthesis genes can also be derived from co-expression data. As biosynthesis genes in the same pathway are often expressed simultaneously, various types of co-expression analyses have emerged as a powerful approach towards the identification of candidate genes. Thus based on one known gene in a biosynthesis pathway, additional genes in the same pathway can be identified. These simple correlation analyses of gene expression values have been replaced by more sophisticated machine learning algorithms [9]. Indeed information about gene expression can be integrated with metabolomics and proteomics data in a multiomics approach. Gene expression can be linked to the presence of metabolites of interest to identify the genes responsible for the corresponding biosynthesis pathway. Once completely resolved, plant biosynthesis pathways will be useful resources to be harnessed for heterologous production in other systems.

With this collection we hope to attract submissions that cover a wide range of topics, including genome sequencing, transcriptomics, comparative genomics, and bioinformatics analyses of medicinal plants.

## Data Availability

Not applicable.
